# Evaluating Cerebral Blood Flow among Patients Experiencing Premenstrual Syndrome with Headache using Duplex Ultrasonography

**DOI:** 10.2174/0115734056326531250529161603

**Published:** 2025-06-24

**Authors:** Pinar Cakmak, Özlem Kosar Can, Ahmet Baki Yagci

**Affiliations:** 1 Department of Radiology, Pamukkale University, Faculty of Medicine, Denizli, Turkey; 2 Department of Obstetrics and Gynecology, Pamukkale University, Faculty of Medicine, Denizli, Turkey

**Keywords:** Duplex ultrasound, Premenstrual syndrome, Headache, Dysmenorrhea, Carotid arteries, Ultrasound

## Abstract

**Introduction:**

This study aimed to demonstrate the relationship between hemodynamic changes in head blood flow and headache during the premenstrual period in patients experiencing premenstrual syndrome.

**Methods:**

Thirty-two female patients experiencing premenstrual headaches were prospectively examined using carotid and vertebral artery duplex ultrasonography during headache episodes in the premenstrual periods and headache-free periods across two consecutive menstrual cycles. The diameters and areas of both the carotid and vertebral arteries, along with systolic and end-diastolic velocities, pulsatility and resistivity indices, and volumetric flow rates, were measured using grayscale imaging. Total head blood flow was determined as the sum of bilateral common carotid artery and vertebral artery flow volumes. Measurements were compared between participants’ premenstrual and menstrual periods.

**Results:**

A statistically significant difference in the diameter of the left external carotid artery was observed between periods with and without headache during the two consecutive menstrual cycles assessed (*p* = 0.030). Left external carotid artery (*p* = 0.019), total external carotid artery (*p* = 0.028), and total head blood volumes (*p* = 0.030) were significantly higher when headache was present during the premenstrual period than when headache was absent.

**Discussion:**

Towards the end of the luteal phase, the total head blood flow and external carotid artery flow were high due to a decrease in peripheral resistance caused by the decline in progesterone and hormonal fluctuations during this period.

**Conclusion:**

Increased flow volume in the external carotid arteries and total head blood flow may be a contributing factor to premenstrual headaches.

## 1 INTRODUCTION

Premenstrual syndrome (PMS) is a condition that involves short-term physiological and psychological symptoms at the end of the menstrual cycle's luteal phase. The physical signs include breast swelling, headache, weakness, and weight gain, and the psychological signs comprise irritability, tension, and depressive mood. These symptoms are found to be severe in 10-15% of women, and 3% of symptoms have been reported to affect the quality of life during this period [1]. Headaches occurring during the premenstrual period have also been defined and categorized as menstrual migraines [2]. A symptom-free interval between the end of menstruation and the ovulation period is essential for excluding other factors that could cause PMS symptoms. A headache during the premenstrual period spans five days, with the highest severity occurring on the menstrual cycle's first day [3].

Among healthy women, no significant differences in the carotid arteries' flow volume have been observed with duplex ultrasonography examination during the menstrual period [4]. Some studies have also reported that women with premenstrual syndrome may experience hormonal disorders in the pathogenesis of PMS during the luteal phase [5]. Although our study is not the first to investigate cerebral blood flow abnormalities among patients with premenstrual syndrome accompanied by a headache, it is a supportive study in which we surmised that progesterone's effect on vascular structures decreases peripheral resistance by affecting the carotid arteries' end-stream, and this effect can be determined with duplex ultrasonography. Thus, the present study aimed to demonstrate the relationship between hemodynamic changes during the premenstrual period and headaches among patients experiencing premenstrual syndrome.

## 2 METHODS

### 2.1 Study Group

This prospective study was conducted between October 2017 and October 2018 at the Obstetrics and Gynaecology Department at Pamukkale University Hospital of Medical Faculty, a tertiary health center hospital in Turkey's Denizli province, after the Institutional Ethics Committee (Pamukkale University Hospital) of the Non-Invasive Clinical Trials approved the protocol (Number 60116787-020/66914) based on the principles of the Declaration of Helsinki. In total, 42 women experiencing PMS symptoms with no history of medication or alcohol use, endocrinological, neoplastic, inflammatory/infectious disorders, or neurological or psychiatric diseases were included in the study. Written informed consent was acquired from each patient before examination. Ten patients were excluded from the study because they did not attend their scheduled second duplex ultrasonography examination, and participants were questioned meticulously about headaches and premenstrual symptoms. The carotid and vertebral arteries of 32 female patients experiencing headaches in their premenstrual periods (age range of 19-35 years; median age 23 years; mean age 24.6 8±5.23 years) were prospectively evaluated by a radiologist with 15 years of ultrasonography experience. All patients were examined twice through carotid and vertebral artery duplex ultrasonography measurements when a headache was present during the premenstrual period and when a headache was absent during two consecutive menstrual cycles.

### 2.2 Ultrasonographic Examination

Duplex ultrasonography measurements were performed with a 9-15 MHz linear probe and the LOGIQ E9 ultrasound device (LOGIQ E9; GE Healthcare, Wauwatosa, WI) by a radiologist who was unaware of each patient's menstrual status. Participants were questioned about cigarette smoking, tea and coffee consumption, and medication ingestion before their ultrasonographic examinations. During both ultrasound examinations, care was taken to ensure that patients had normotensive blood pressure and sinus rhythms. The room where the ultrasonographic examinations were performed was kept dark, quiet, and at 22-24°C. Participants' ultrasonographic examinations took place after they had rested in a supine position for at least 10-15 minutes. Also, care was taken during the ultrasonographic examinations to use adequate gel volumes and apply gentle pressure to prevent artefacts. Common carotid artery (CCA) measurements were obtained 1.5-2 cm proximally to the carotid bulb, while internal carotid artery (ICA) and external carotid artery (ECA) measurements were obtained 1.5 cm distally to the carotid bulb in the longitudinal and transverse planes. Vertebral artery (VA) measurements were obtained from the V2 segment at the level of the C4 and C5 vertebrae's transverse processes, and diameter and area measurements were obtained using grayscale images and recorded for bilateral CCA, ICA, ECA, and VA. The diameter measurements of all vessels were obtained in the wall-to-wall axial and longitudinal planes, and their averages were calculated.

Duplex ultrasonography spectral examination was performed by placing the sample volume at the central portion of the examined vessel. The sampling interval was 1 mm, and the insonation angle was 60°. During the spectral examination, the signals emitted by the patients' vasculature were obtained without artefacts for at least three consecutive cardiac cycles, and the measurements were obtained from a frozen screen. The participants' systolic and end-diastolic velocities, pulsatility and resistivity indices, and flow volume values were measured three times and averaged by means of the ultrasonography device software. Total head blood volume was determined as the sum of bilateral CCA and VA volume. All sonographic measurements were compared with participants' demographic characteristics, and ultrasonographic measurements obtained during the patients' premenstrual and menstrual periods were compared.

### 2.3 Statistical Analysis

Statistical analyses were performed using statistical software (SPSS 21 for Windows, Chicago, IL). The descriptive statistics used included mean±standard deviation for continuous variables and percentages for categorical variables. A Wilcoxon signed-rank test was conducted to compare ultrasonographic measurements, and *p* < 0.05 was considered statistically significant.

## 3 RESULTS

Thirty-two participants experienced headaches during their premenstrual periods. A statistically significant difference was observed between the diameter of the left ECA measured while a premenstrual headache was present, and the corresponding measurement was obtained between two consecutive menstrual cycles (*p* = 0.030) (Fig. **1a** and **b**). No significant differences were observed in the measurements of bilateral CCA, ICA, VA, right ECA, and peak systolic velocity (PSV), end-diastolic velocity (EDV), resistive index (RI), or pulsatility index (PI) between the headache-presence and headache-absence periods (Table **1**). Total head blood volume was calculated as the sum of both CCA and VA blood volumes. Left ICA (*p* = 0.036), left ECA (*p* = 0.019), total ECA (*p* = 0.028), and total head blood volume (*p* = 0.030) were significantly higher when a headache was present during the premenstrual period than when a headache was absent (Figs. **2a** and **b**). However, no significant differences were observed between bilateral VA, bilateral CCA, right ICA, or total cerebral blood volume (Table **2**).

## 4 DISCUSSION

The International Classification of Headache Disorders, 3^rd^ edition (ICHD 3) defines menstrual migraine as migraine attacks that occur during a two-day period predating the menstruation cycle and a three-day period after the menstruation cycle [1]. ICHD-3 definitions also categorize menstrual migraine without aura, pure menstrual migraine (PMM), and menstruation-related migraine (MRM). For women with PMM, attacks only occur during the menstrual period, and headaches should be present during at least three consecutive menstrual cycles. Vetvik *et al.* reported the menstrual migraine's prevalence at 20% among women with migraines when these diagnostic criteria were used [6]. A study by Martin *et al.* assessed the relationship between ovarian hormones and menstrual migraine, concluding that migraine pathogenesis involves hormonal fluctuations, estrogen withdrawal, and reduced progesterone toward the end of the luteal phase [7, 8]. Different studies have also found a positive correlation between urinary progesterone metabolites and headaches during the luteal phase [5, 9]. One reason for this correlation is that estrogen withdrawal increases the trigeminal afferents' excitability and the functions of neurotransmitter systems and microglia, which may trigger menstrual migraine headaches [10]. However, our study did not assess serum hormone levels among women with PMS.

Assessing blood flow velocity and volume in the vertebral and carotid arteries through duplex ultrasonography is an important parameter in diagnosing and following up on ischemic cerebrovascular disease. The literature has reported blood flow volumes of the extracranial carotid and vertebral arteries among patients and healthy groups using duplex ultrasonography [11-16]. Further research has examined the relationship between arterial dilation or hyperperfusion and headaches, but a clear relationship between these processes has not yet been established. The present study assessed the carotid and vertebral arteries' velocity, flow volume, and resistivity and pulsatility indices among female participants, finding a significant increase in left ICA, left ECA, total ECA, and total head blood flow volumes when patients experienced headaches during the premenstrual period. Moreover, this study found no significant differences in other vascular structures' flow volumes between the symptomatic and asymptomatic periods. Additionally, no significant differences in RI, PI, or velocity values were observed between these periods.

Krejza *et al.* examined carotid arteries with duplex ultrasonography before and after administering acetazolamide (ACE) during the menstrual cycle [4]. In their study, they did not observe a significant change in the carotid arteries during menstrual cycles without ACE administration but found a significant increase in CCA and right ICA velocity on the fifth and 26^th^ days following ACE administration. Their study revealed a velocity reduction in the ECAs after ACE administration. The present study found carotid and vertebral artery flow volume values similar to the literature's corresponding values when a headache was absent during two consecutive menstrual cycles. Additionally, this study found significant increases in left ECA diameter, left ICA, left ECA, total ECA, and total head blood flow volumes when a headache was present during the premenstrual period, while right ICA and vertebral artery volumes remained consistent during the premenstrual headache period. Also, a premenstrual headache may be caused by increased blood flow volume in the ECAs. Toward the end of the luteal phase, total head blood and ECA flow were high due to a decrease in peripheral resistance caused by the decline in progesterone and hormonal fluctuations during this period. The clinical significance of this study is that the results can support treatment planning for headaches and other PMS symptoms.

This study presented some limitations. First, some patients refused repeated ultrasonographic examinations and meticulous questioning about headaches and premenstrual symptoms, and the study was completed with only 32 participants. Second, patients experiencing PMS who also had headaches during the study period comprised the study group. Additionally, other radiological imaging processes were not performed to assess the patients' headaches during the study period. Third, all patients were evaluated by a single radiologist. Ultrasonography is a user-dependent modality, and no assessment of inter-observer variability was performed in this study. Another limitation was that the sample volume used for the study's spectral analysis was not obtained according to the exact diameters of the measured vessels. The sample volume was placed at the standard diameter and central location of the measurement for each patient before and after menstruation. The study was limited to women in Turkey and did not consider people of other races or regions. Finally, PMS patients' luteal-phase and midcycle progesterone and estrogen levels, and pCO_2_ blood concentrations were not determined.

## CONCLUSION

This study found that flow volumes in the left internal carotid artery, left external carotid artery, total external carotid artery, and total head blood volume were significantly higher when a headache was present during the premenstrual period compared to when a headache was absent. We conclude that increased flow volumes in the external carotid arteries and total head blood volume may be one of the causes of premenstrual headaches. Moreover, the duplex ultrasonography data from this study may contribute to future research on therapeutic approaches to premenstrual headaches.

## Figures and Tables

**Fig. (1a):**
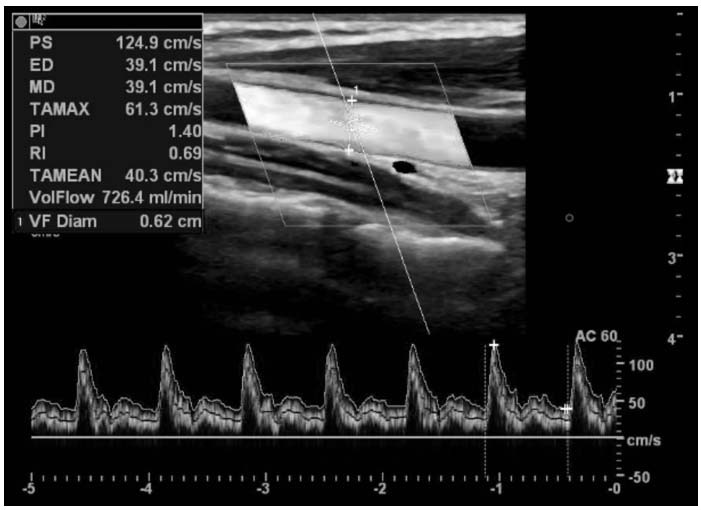
Left CCA duplex ultrasonography image, showing PS, ED, PI, RI, vessel diameter, and flow volume values during the premenstrual headache period.

**Fig. (1b):**
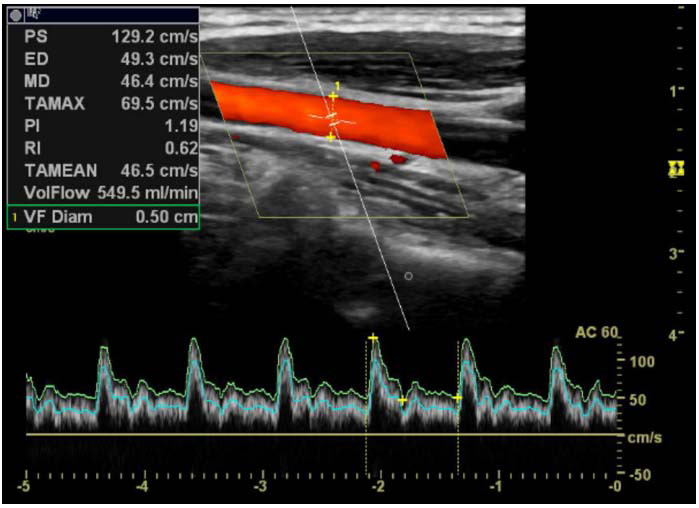
Left CCA duplex ultrasonography image, showing PS, ED, PI, RI, vessel diameter, and flow volume values during the headache-free period.

**Fig. (2a):**
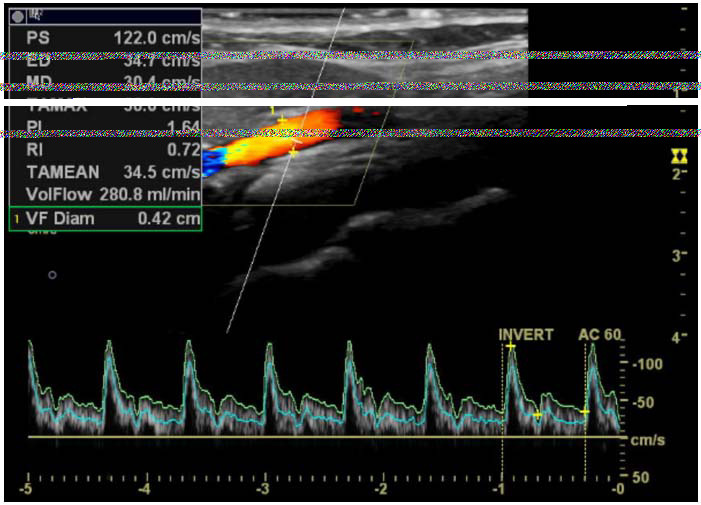
Left ECA duplex ultrasonography image, showing PS, ED, PI, RI, vessel diameter, and flow volume values during the premenstrual headache period.

**Fig. (2b):**
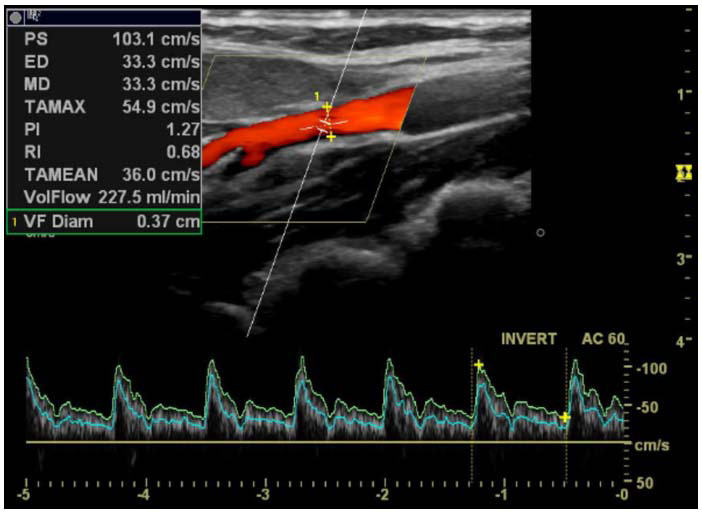
Left ECA duplex ultrasonography image, showing PS, ED, PI, RI, vessel diameter, and flow volume values in the headache-free period.

**Table 1:** Duplex ultrasonography measurements of the carotid and vertebral arteries.

		**Diameter (mm)**			**PSV (cm/sec)**			**EDV (cm/sec)**			**RI**			**PI**		
**Headache**		**Positive (PMS)**	**Negative (IMP)**	***P- value***	**Positive (PMS)**	**Negative (IMP)**	***P- value***	**Positive (PMS)**	**Negative (IMP)**	***P- value***	**Positive (PMS)**	**Negative (IMP)**	***P- value***	**Positive (PMS)**	**Negative (IMP)**	***P- value***
**Right**	**CCA**	5.38±0.49	5.6±0.55	0.177	104.8±11.97	103.71±27.43	0.808	34.32±4.81	33.07±5.9	0.212	0.69±0.06	0.68±0.03	0.241	1.47±0.31	1.38±0.18	0.082
	**ICA**	4.33±0.56	4.32±0.47	0.920	95.38±11.93	98.53±13.33	0.590	42.01±5.72	43.04±6.88	0.626	0.57±0.04	0.56±0.07	0.856	0.98±0.28	0.91±0.21	0.590
	**ECA**	3.61±0.46	3.53±0.44	0.161	98.75±12.55	98.7±17.15	0,758	22.1±7.01	22.78±3.81	0.363	0.78±0.08	0.76±0.04	0.239	1.93±0.5	1.9±0.36	0.661
	**VA**	3.03±0.33	3.03±0.32	0.955	52.97±9.35	55.86±7.88	0.079	19.62±8.62	23.19±4.21	0.066	0.63±0.16	0.58±0.07	0.109	1.1±0.42	0.97±0.22	0.144
**Left**	**CCA**	5.42±0.59	5.32±0.59	0.468	108.17±9.69	104.28±11.01	0.082	35.18±4.9	34.17±4	0.433	0.67±0.04	0.67±0.05	0.944	1.36±0.18	1.4±0.2	0.398
	**ICA**	4.48±0.64	4.19±0.55	0.073	90.27±13.25	90.84±12.27	0.649	41.66±8.21	40.52±5.98	0.741	0.54±0.07	0.55±0.08	0.711	0.88±0.25	0.9±0.22	0.601
	**ECA**	**3.59±0.44**	**3.34±0.42**	**0.030**	91.02±14.35	87.26±12.17	0.266	23.46±6.79	21.54±7.9	0.114	0.76±0.08	0.76±0.1	0.848	1.76±0.5	1.8±0.49	0.673
	**VA**	3.34±0.52	3.3±0.38	0.659	58.57±9.2	62.11±12.57	**0.050**	24.04±7.19	25.39±5.14	0.696	0.59±0.12	0.58±0.07	0.751	1.03±0.35	1.02±0.23	0.520

**Abbreviations:** PMS: Premenstrual syndrome, IMP: Intermenstrual period, CCA: Common carotid artery, ECA: External carotid artery, ICA: İnternal carotid artery, VA: Vertebral artery, PSV: Peak systolic velocity, EDV: End-diastolic velocity, RI: Resistive index.

**Table 2:** Blood flow volumes during headache and asymptomatic intermenstrual period.

**Volume (ml/min)**	**Headache Negative** **Intermenstrual Period**	**Headache Positive** **Premenstrual Syndrome**	***p***
**Right CCA**	454.54±103.34	513.1±134.92	0.095
**Left CCA**	425.98±115.21	483.53±130.59	0.108
**Right ICA**	361.89±74.33	351.71±97.76	0.485
**Left ICA**	316.61±78.85	363.75±98.21	**0.036**
**Right VA**	99.78±28.3	89.85±29.28	0.104
**Left VA**	123.19±46.07	129.54±54.93	0.733
**Right ECA**	160.90±55.17	171.42±54.58	0.372
**Left ECA**	132.91±64.18	162.63±64.72	**0.019**
**Total ECA**	293.82±99.36	334.05±105.0	**0.028**
**Total cerebral blood flow**	901.47±131.43	934.85±210.36	0.390
**Total head blood flow**	1103.48±191.19	1216.01±194.47	**0.030**

**Abbreviations:** CCA: Common carotid artery, ECA: External carotid artery, ICA: Internal carotid artery, VA: Vertebral artery.

## Data Availability

The data and supportive information are available within the article.

## LIST OF ABBREVIATIONS

PSV: Peak systolic velocity
EDV: End-diastolic velocity
RI: Resistive index
PI: Pulsatility index
VA: Vertebral artery
ICA: Internal carotid artery
ECA: External carotid artery
CCA: Common carotid artery

## STANDARDS OF REPORTING

STROBE guidelines were followed.
